# The optimal spacing interval between principal shelterbelts of the farm-shelter forest network

**DOI:** 10.1007/s11356-021-17272-1

**Published:** 2022-01-05

**Authors:** Qinming Sun, Bo Zheng, Tong Liu, Lekui Zhu, Xiaoran Hao, Zhiquan Han

**Affiliations:** 1 Agricultural College, Shihezi University, Shihezi, 832003 China; 2 College of Life Sciences, Shihezi University, Shihezi, 832003 China; 3 Key Laboratory of Special Fruits & Vegetables Cultivation Physiology and Germplasm Resources Utilization of Xinjiang Production and Construction Corps, Shihezi, 832003 China

**Keywords:** Farm-shelter forest network, Spacing interval, Structure configuration, Farmland shelterbelt, Windbreak effect, Wind erosion

## Abstract

The farm-shelter forest network is a complex grid protection system, with a windbreak that is distinctly different from that of the single shelterbelt. We selected the farm-shelter forest network of a jujube field in the Tarim Basin of northwest China and used a combination of field measurements and wind tunnel tests to determine the optimal spacing interval between principal shelterbelts. The wind speed reductive curve of the farm-shelter forest network showed a gradual wind speed tendency to stability. Therefore, a model was established based on the energy transfer balance between the upper and the lower airflows for a steady wind speed. The prediction error of the model was found to be < 1%. The model results indicated that increasing the spacing interval between principal shelterbelts from 10 to 20 *H*, where *H* is the shelterbelt height, maintained more than 70% of the windbreak effect of the farm-shelter forest network. If the spacing interval between principal shelterbelts were to be increased from 10 to 20 *H*, the jujube planting area would be increased by 0.54%. Therefore, a thorough consideration of the windbreak effect of each shelterbelt, the synergistic effects of shelterbelts, the windbreak effects of tall crops, and the effects of temperature and humidity in farm-shelter forest networks indicates that increasing the spacing interval will not only maintain the windbreak effect, but it will also reduce the side effects of shelterbelts, increase the planting area, favor mechanized operation, and improve planting efficiency.

## Introduction

Farm-shelter forest networks play an important role in producing stable and high crop yields, stabilizing the farmland ecosystem, and improving the microclimate of agricultural fields, primarily through the reduction of windstorm disasters (Kowalchuk and Jong, 1995; Zheng et al., 2016a, b; Zhu et al., 2017; He et al., 2017). The basic principles of wind control indicate that windbreaks planted or placed in the path of air flow act as surface barriers, causing an upward diversion of the air current, and this diversion is accompanied by a drag on the wind at certain heights of the windbreaks (Woodruff, 1956; Maki, 1985; Zhu, 2013). These combined effects lessen the force of wind on the original ground surface, lower the prevailing surface velocity, and create an area of relatively calm airflow within the zone of influence of the windbreaks. In the case of windbreaks, protection can be achieved by establishing a system of properly oriented windbreaks. The most common design parameter for a system of windbreaks is the spacing interval (Cao, 1983; Zhu, 2013).

The construction of farmland shelterbelts has been studied extensively. In particular, the windbreak effect has been investigated in terms of efficient protection distance (Ma et al., 2010; Ferreira and Lambert, 2011; Dong et al., 2011; Wu, et al., 2013; Dang et al., 2014). However, since the farm-shelter forest network is a complex grid protection system, there has been little research on the construction of networks composed of multiple shelterbelts, especially on the optimal spacing interval between principal shelterbelts. The overall windbreak effect of a farm-shelter forest network depends not only on the windbreak effect of each shelterbelt, but also on the synergistic effects of shelterbelts, the windbreak effects of tall target crops, and the effects of temperature and humidity (Cao, 1983; Zhu, 2013).

Synergistic effects among shelterbelts have been confirmed. Airflow velocity has been found to decrease after passing through the first shelterbelt in a farm-shelter forest network. Before the wind speed can recover to its initial intensity, it is affected by a second shelterbelt, such that the wind within a forest network continues to maintain a low speed, and it eventually tends to stabilize (Maki, 1982; Cao, 1983; Zuo et al., 2018). Moreover, the airflow velocity actually decreases between the first and second shelterbelts. On the basis of previous research, Li and Sherman (2015) reported that the protective distance increased with increasing numbers of shelterbelts having the same porosity. Via wind tunnel simulation experiments, Bao (2015) discovered that the effective protected area beyond the second shelterbelt in a farm-shelter forest network could be increased using multilayer shelterbelts.

The protective effect of farm-shelter forest networks is not only affected by changes to the structure of the network, but it is also closely related to the atmospheric properties within it (Cao, 1983). Shelterbelts not only reduce the wind speed, but they also affect the vertical variation of water vapor and the spatial distribution of heat (Livesley et al., 2004; Chirwa et al., 2007; Cayan et al., 2010), leading to changes in soil water evaporation and plant transpiration in farm-shelter forest networks. In particular, shelterbelts can significantly reduce the temperature by 40–60%, while also increasing the humidity and soil water content within the forest on a regional scale (Kamal, 2014; Zhuang et al., 2017). Therefore, the water vapor content of the air within the forest is increased by shelterbelts, as is the viscous resistance. Finally, wind speed is also affected by farm-shelter forest networks.

Farm-shelter forest networks are designed to protect target crops. However, plant heights and areal changes of target crops are associated with varying roughness (Kustas et al., 2005; Ding, 2010; Wu et al., 2016; Vanderwende and Lundquist, 2016), and transpiration (Sun et al., 2011, 2016), which can affect wind speed. Zheng et al. (2016a, 2016b) reported that, just in terms of the protective effect, efficiently spaced shelterbelts could decrease the shelterbelt areas and increase crop areas, thereby enhancing the economic benefits of crops. Li et al. (2015) reported that crops had the greatest potential for reducing the flux of windblown dust, while red date orchards and cotton fields had the lowest potential for dust flux (due to their highest aerodynamic roughness). Accordingly, when thoroughly considering the protective effect of crops, is it possible to further reduce the spacing interval between principal shelterbelts, decrease the shelterbelt area, and promote the economic benefits of crops?

The study site of this investigation was the farm-shelter forest network of a jujube field in the Tarim Basin, China, which is a region that experiences some of the most severe wind-sand disasters in the world. The characteristics of the wind speed variations in the farm-shelter forest network were analyzed using field measurements and wind tunnel tests. An arrangement model for the farm-shelter forest network was established based on the above results, and the optimal spacing interval between principal shelterbelts was determined. Given the rapid development of modern intensive agriculture, precision agriculture, and mechanized agriculture, this research has important theoretical and practical significance for the construction of farm-shelter forest networks that ensure the windbreak effect, while also reducing the side effects of shelterbelts (Kort, 1988; Qiao et al., 2016) and improving planting efficiency.

## Materials and methods

### Study area

**Fig. 1:**
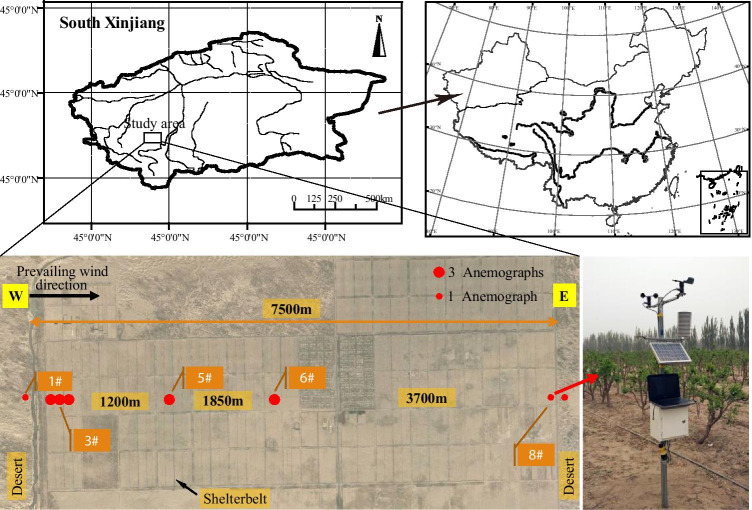
Location of the study area and distribution of anemographs. There were nine observation points in the study area. The observation points were situated along the prevailing wind direction in the forest. In order to determine the role of shelterbelts in the western farm-shelter forest network, the observation points were densely situated in the western section, and sparsely positioned in the eastern section. Sample point 1 in the outside forest was set as the control

The study area (37°12′29″–37°24′51.5″N, 79°14′57″–79°22′25″E, at elevations of 1304–1397 m) is located in Farm 224, Hotan Prefecture, in the southern Tarim Basin of Xinjiang, China (Fig. 1). The Tarim Basin is one of the primary dust sources in the arid and semiarid regions of the world. Dust emitted from the Tarim Basin can be transported by winds across Asia and the Pacific Ocean (Yu et al., 2012). The study area (21.8 × 9.2 km) is surrounded by deserts, including the Gobi, and its elevation is greater in the south than in the north. The arid desert climate of the study area is characterized by a mean annual precipitation of 32.1 mm, an annual potential evaporation of 2563.9 mm, an annual mean temperature of 12.2 °C, a maximum temperature of 42.7 °C, and a minimum temperature of -23.7 °C. Wind-sand events are the prevalent type of meteorological disaster in the study area, and they generally include high winds, dust storms, dust clouds, and hot, dry air in the spring, summer, and autumn. Gale events occur an average of 11.5 times per year, and airborne dust events occur 200 days/year. The prevailing wind direction in the spring is west-northwest, with a maximum wind frequency of 41.7% (The National Meteorological Information Center of the China Meteorological Administration provided meteorological data). The study area, with jujube farming as the leading industry and drip irrigation as the main water application method, has an extensive farm-shelter forest network.

### Structural factors of the farm-shelter forest network

Based on shelterbelt length and other indicators, we selected different types of shelterbelt structures and surveyed these shelterbelts as well as the jujube field using the methods described by Guan et al. (2002). The direction of the principal shelterbelts was north–south in this study, because the prevailing wind direction was east–west. The shelterbelts perpendicular to the prevailing wind direction were defined as the principal shelterbelts, and the shelterbelts parallel to the prevailing wind direction were defined as the assistant shelterbelts in this study. Specifically, a 100-m section was selected near the center of the principal shelterbelt as the study sample; dead trees were tallied to calculate survival rates; the perimeter method was used to measure *DBH* (diameter at breast height); crown width was estimated visually; mean height, mean height under branches, and mean crown height were measured digitally; and tree species, row number, row spacing, in-row spacing, lengths, and widths were recorded. For rapid and accurate quantitative measurement of shelterbelt porosity, windbreak porosity was measured digitally.

### Wind observations in the farm-shelter forest network

A farm-shelter forest network, 7.5 km long from east to west, in which trees grew and were preserved well, was selected as the study area. The observation points were situated along the prevailing wind direction in the forest. Anemographs were used to determine wind speed, wind direction, temperature, and humidity at different locations. In order to determine the role of shelterbelts in the western farm-shelter forest network, the observation points were densely situated in the western section, and sparsely positioned in the eastern section. In addition, two observation points were placed outside the farm-shelter forest network to serve as references (Fig. 1).

Wind speed and wind direction were recorded from the germination stage to the fruit harvest of the jujube crop (April 23–October 26, 2016). Wind speed and wind direction data were collected with sampling rate of once every 2 min using an automatic wind speed recorder (XE48/YM-24); 2-min average windspeed and direction can be read directly from the instruments. The measurement height was 2 m. Sample point 1 in the outside forest was set as the control. The layout of the wind speed recorders is shown in Fig. 1. The wind profile in the farm-shelter forest network was determined using a field vertical anemometer (heights of 0.2 m, 0.5 m, 1.5 m, and 2.5 m). These data were primarily used to verify the wind profile in the wind tunnel.

### Wind tunnel simulation experiment of the wind speed characteristics in the farm-shelter forest network

**Fig. 2:**
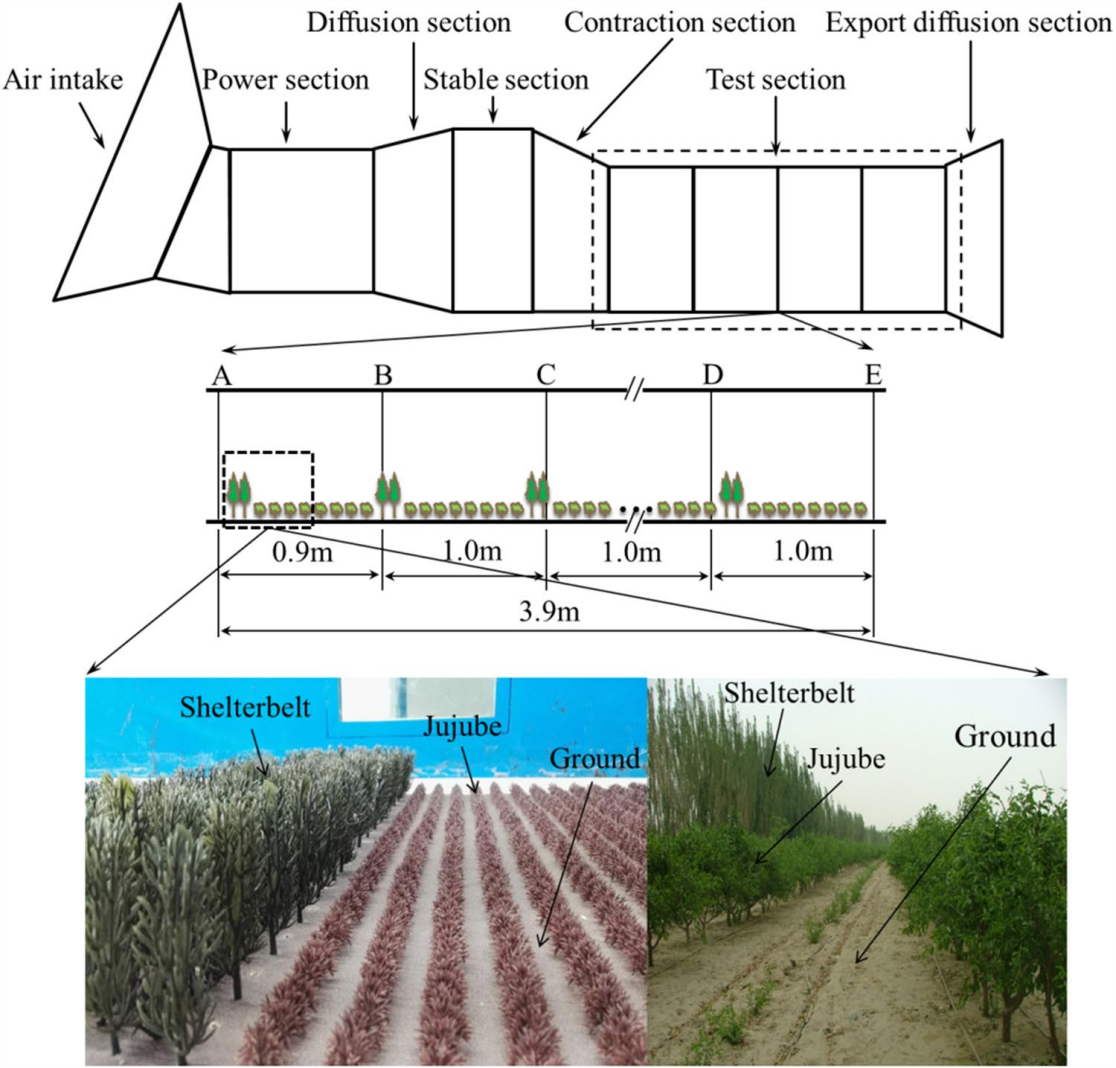
Schematic of wind tunnel structure, test section, and shelterbelt and jujube model

The wind tunnel simulation test was carried out in a movable environmental wind tunnel (16.2 m; Fig. 2) at the Xinjiang Institute of Ecology and Geography, Chinese Academy of Sciences. The test section of the wind tunnel was rectangular; its length was 8 m, width was 1.3 m, and height was 1 m, with an aspect ratio is 1:3. The wind speed along the axis of the test section could be adjusted continuously within a range of 1–25 m/s. The stability coefficient of the airflow in the wind tunnel was < 1%, the lateral nonuniformity was < 2.5%, the turbulence intensity was ~ 1%, and the boundary layers of the bottom and the side were 15 cm and 10 cm, respectively. Transverse sections at 0 m, 0.9 m, 1.9 m, 2.9 m, and 3.9 m behind the entrance of the work section were selected as the test sections; the locations are shown in Fig. 2(A–E). There were 10 measuring points on each test transverse section. Ten pitot tubes were mounted on brackets at heights of 0.5 cm, 1.0 cm, 1.5 cm, 2.0 cm, 5.0 cm, 8.0 cm, 10.0 cm, 15.0 cm, 30.0 cm, and 50.0 cm.

The shelterbelt and jujube models were made of soft plastic trees based on the similarity theory and actual investigations of tree height and porosity in wind tunnel experiments. The height of the shelterbelt model was 7 cm, and the height of jujube tree model was 1.5 cm. The shelterbelt and jujube models were zoomed out by the same scale, and the model to real object ratio was 1:143. The surface of the farmland was simulated with 125-μm sand paper (Fig. 2).

The major factors influencing a shelterbelt’s windbreak effect are its height, porosity, width, section shape, angle between it and the wind direction, principal shelterbelt spacing intervals, and other factors (Zhu, 2013). Four wind speeds were set in the wind tunnel simulation: 8 m/s, 10 m/s, 12 m/s, and 14 m/s. The effect of jujube trees on wind speed in the shelterbelts was analyzed by including and removing the jujube trees in the wind tunnel simulation. The effect of crops on the wind relief provided by shelterbelts was further analyzed. By increasing the number of shelterbelts in the same area (there were a total of 16 rows of forest belts and the sum of the belt spacing was 30 *H*), the cumulative effect of multilayer shelterbelts was analyzed. The maximum number of shelterbelts could be set to 4 since the length of the wind tunnel test section was 8 m.

The design of the farm-shelter forest network in the wind tunnel simulation experiment is shown in Table 1.

**Table 1:** Design of the farm-shelter forest network in the wind tunnel simulation experiment

Number	Width of core shelterbelt/row	Shelterbelt spacing (1/H)	Shelterbelt 1 in farmland/row	Shelterbelt spacing (2/H)	Shelterbelt 2 in farmland/row
1	4	12	6	18	6
2	6	16	5	14	5
3	8	20	4	10	4
4	10	10	3	20	3
5	12	14	2	16	2
6	12	18	1	12	1
7	12	18	2	12	2
8	8	–	–	–	–
9	8	20	0.4	–	–
10 (CK)	–	–	–	–	–

### Construction of the farmland shelterbelt allocation model

#### Surface wind field structure in the farm-shelter forest network

**Fig. 3:**
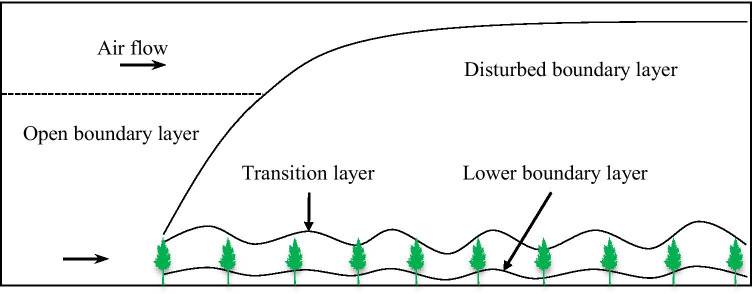
Boundary layer of the farm-shelter forest network

Boundary layer of the farm-shelter forest network is shown in Fig. 3.

According to previous research (Zhu and Zhou, 1993), airflow with a certain velocity (initial velocity *u*_0_) enters from a smooth surface to the farm-shelter forest network, then plateaus after a period of time, forming stable boundary layers. There are three stable boundary layers in the farm-shelter forest network: the open boundary layer, lower boundary layer, and disturbed boundary layer. The variation of wind speed with height follows the logarithm law in the three layers.

For neutral conditions, the form of the wind velocity profile in the wild is as follows:

1 $${u}_{0} =\frac{{ u}_{*0}}{k}ln\frac{Z}{{Z}_{0}},$$

where *u*_0_ is the wind velocity, u_*0_ is the dynamic speed, *z*_0_ is the roughness, and *z* is measurement height.

The form of the wind velocity profile in the lower boundary layer is as follows:

2 $${u}^{^{\prime}}= \frac{{u}_{*}^{^{\prime}}}{k}ln\frac{Z}{{z}_{0}^{\prime}{^{\prime}}}$$

where *u*', u_*_', and *z*_*0*_*"* are the velocity, dynamic speed, and roughness in the lower boundary layer, respectively.

The form of the wind velocity profile in the disturbed boundary layer is as follows:

3 $$u=\frac{{u}_{*}}{k}ln\frac{Z}{{z}_{0}^{{\prime}}}$$

where *u*, u_*_, and *z*_*0*_*'* are the velocity, dynamic speed, and roughness in the disturbed boundary layer, respectively.

#### Energy balance theory

The model was based on the energy transfer balance between the upper and lower airflows for a steady wind speed. Friction was increased by surface roughness when the airflow entered the forest area from the flat and homogeneous wilderness area. As a result, the momentum flux transmitted from the upper layer to the lower layer increased to supplement the kinetic energy consumed by the lower airflow, and equilibrium was eventually reached. The momentum flux of a single forest grid in the disturbed boundary layer was as follows:

4 $$M={L}_{1}{L}_{2}\rho {u}_{*}^{2}$$

where *L*_1_ is the length of the principal shelterbelt, *L*_2_ is the length of the assistant shelterbelt (the width of the shelterbelt is neglected here; *L*_2_ is the distance between principal shelterbelts), and *ρ* is the air density.

The surface friction was composed of 2 parts: the resistance of the principal shelterbelt and the resistance of the forest area, as follows:

5 $$M={\tau }_{1}+{\tau }_{2}$$

where *τ*_1_ is the resistance of the principal shelterbelt and *τ*_2_ is the resistance of the forest area.

#### Resistance of the principal shelterbelt

Based on the flow resistance formula of fluid mechanics proposed by Newton in 1726 (Zhu and Zhou, 1993; Ding, 2010), let *H* be the shelterbelt height, *L*_1_ be the principal shelterbelt length, *L*_2_ be the secondary shelterbelt length, *α* be the shelterbelt ventilation coefficient, *β* be the shelterbelt porosity, *ω* be the angle between the wind direction and the shelterbelt, and *ρ* be the air density. The resistance of the principal shelterbelt *τ*_1_ was as follows:

6 $${\tau }_{1}={F}_{D}H{L}_{1}\mathit{sin}\omega =\frac{1}{2}{C}_{d}\rho {{u}_{e}}^{2}H{L}_{1}\mathit{sin}\omega$$

*C*_*d*_ is the drag coefficient in the formula; its calculation is from Zhu (2013):

7 $${C}_{d}=1.63{\left(1-\alpha \right)}^{0.55}$$

*α* is the ventilation coefficient in the formula; its calculation is from Ren et al. (2013):

8 $$\alpha ={\beta }^{0.55}$$

*u*_*e*_ is the resistance wind speed of the shelterbelt. The geometric mean of the wind speed in the 5–10 H range in front of the shelterbelt in the farm-shelter forest network is directly proportional to *u*_*e*0_. *u*_*e*0_ is the initial wind speed in front of the farm-shelter forest network.

9 $${u}_{e}={r}_{e}{u}_{e0}={r}_{e}\frac{{u}_{*0}}{K}\mathit{ln}\frac{H}{e{z}_{0}}$$

*r*_*e*_ is the coefficient of wind speed reduction of the shelterbelt; its calculation is from Zhu and Zhou (1993):

10 $${r}_{e}=0.179\mathrm{ln}\left(\frac{x}{H}-5\right)+0.288$$

where *x* is the horizontal distance from the shelterbelt.

#### Resistance of one grid of the farm-shelter forest network

The formula is as follows:

11 $${\tau }_{2}={L}_{1}{L}_{2}\rho {{u}_{z}}^{2}$$

where *u*_*z*_ is the dynamic speed in the lower boundary layer, which decreases with increasing roughness in the farm-shelter forest network.

12 $${u}_{z}=\left({r}_{z}+{r}_{s}\right){u}_{*0}$$

where *r*_*z*_ is the coefficient of wind speed reduction in the farm-shelter forest network, which is affected by the surface roughness caused by shelterbelts and crops. Based on Formulas (13) and (14), which were obtained from previous research (Zhu and Zhou, 1993), the empirical curve associated with the coefficient of wind speed reduction and relative wind speed was calculated. Formula (15) was as follows:

13 $$\frac{u}{{u}_{0}}=\frac{{u}_{*}}{{u}_{*0}}\left(1-\frac{\mathrm{ln}A}{\mathrm{ln}\left(Z/{Z}_{0}\right)}\right)$$

14 $$A=\frac{{Z}_{0}^{{\prime}}}{{Z}_{0}}=3.341\bullet {\left(\frac{{u}_{*}}{{u}_{*0}}\right)}^{4}-2.341$$

15 $${r}_{z}=-2.3775{v}^{2}-0.2332v+3.5782$$

where *v* is the relative wind speed, *v* = *v*_1_ + *v*_*2*_.

16 $${v}_{1}=\left(-0.228\mathrm{ln}s+6.85\right)/{u}_{0}$$

where *v*_1_ is the relative wind speed behind the first shelterbelt in the farm-shelter forest network within the study area. The calculation of *v*_1_ consisted of 2 steps: first, the curve of the wind speed change with area was obtained from the field observation experiment, then the relative wind speed was corrected using the wind tunnel experiment.

*s* is the length of the farm-shelter forest network: $s=\sum_{i=1}^{n}{X}_{i}$, where *X* is the spacing interval between principal shelterbelts, and *n* is the number of spacing intervals.

*v*_2_ is the standardized relative wind speed. The relative wind speed for different spacing intervals was standardized using the wind speed of the actual shelterbelt spacing (10 H). *v*_*2*_ was then obtained by averaging the above results.

17 $${v}_{2}=\left[\left({f}_{f}\left({X}_{1}\right)-{f}_{f}\left({X}_{s}\right)\right)+\sum\nolimits_{i=2}^{n}\left({f}_{s}\left({X}_{i}\right)-{f}_{s}\left({X}_{s}\right)\right)\right]/n$$

${f}_{f}(X)=-0.066\mathrm{ln}X+0.241$: The variation of mean relative wind speed with spacing at grid 1.

${f}_{s}(X)=-0.061\mathrm{ln}X+0.2189$: The variation of mean relative wind speed with spacing at grid 2, 3, …, *n*.

#### Coefficient of wind speed reduction by moisture

The guiding concept is that viscous resistance by the air is increased by the moisture originating from vegetation evapotranspiration in the farmland shelterbelt (Zhuang et al., 2017; Sun et al., 2016). The property of fluid viscosity demonstrates that the wind speed is proportional to the viscosity:

18 $${r}_{s}=\frac{{u}_{*}}{{u}_{*0}}=\frac{\left({\mu }_{s}-{\mu }_{k}\right)\bullet RH}{{\mu }_{k}}$$

where *μ*_*k*_ is the viscosity of dry air; in this study, its value was 1.83 × 10^−5^ Pa·s at 20 °C. *μ*_*s*_ is the viscosity of water vapor; its value is 2.31 × 10^−5^ Pa·s at the critical temperature of water vapor, 374.15 °C under 0.1 MPa of pressure. *RH* is the relative humidity.

#### Spacing interval between principal shelterbelts

The windbreak effect (*E*) was then calculated based on Eqs. (4)–(18):

19 $$\begin{array}{c}E=1-\frac{u}{{u}_{0}}\\ =1-\left(1-\frac{\mathrm{ln}A}{\mathrm{ln}\left(Z/{Z}_{0}\right)}\right)\bullet {\left[0.5\frac{1}{{k}^{2}}\bullet H\bullet {\left(\mathrm{ln}\frac{H}{e{Z}_{0}}\right)}^{2}\bullet \frac{{r}_{e}^{2}\bullet {C}_{d}\bullet \mathrm{sin}\omega }{{L}_{2}}+{\left({r}_{Z}+{r}_{S}\right)}^{2}\right]}^\frac{1}{2}\end{array}$$

The windbreak effect (*E*) was altered because the shelterbelt spacing (*L*_2_) was altered. By analyzing the damage threshold wind speeds for different crops, the basic windbreak effect values for different crop damage threshold wind speeds were determined, and the optimal spacing intervals between the principal shelterbelts of different target crops were finally obtained.

## Results

### Analysis of wind variation characteristics in the farm-shelter forest network

**Fig. 4:**
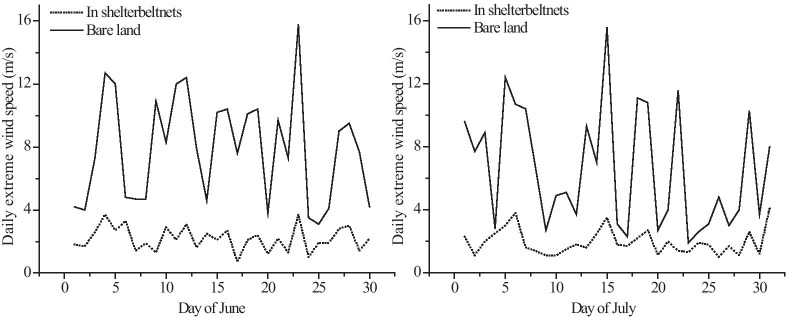
Wind speed measurements of the farm-shelter forest network in June and July, 2016

The wind speed observations measured during the germination to harvest period of the jujube crop revealed that many extreme wind events occurred. Extreme wind events with maximum instantaneous wind speeds higher than 10 m/s occurred approximately 15 times in the study area in June and July, 2016 (Fig. 4). In the two extreme wind events of June 23 and July 15, the wilderness maximum instantaneous wind speed outside the farm-shelter forest network exceeded 14–16 m/s, while the wind speed within the farm-shelter forest network was only ~ 4 m/s, demonstrating that the protective effect of the farm-shelter forest network was quite pronounced. The gales occurring on June 23 and July 15 were selected for study. Their average wind speeds exceeded 10 m/s and they lasted more than 2 h. These events were selected because the hazard threshold wind speed of the target jujube crop is 6.9 m/s (Zhu et al., 2016). If the wind speed was too low in the wilderness, the influence of the wind speed would be insignificant due to the existence of the farm-shelter forest network.

**Fig. 5:**
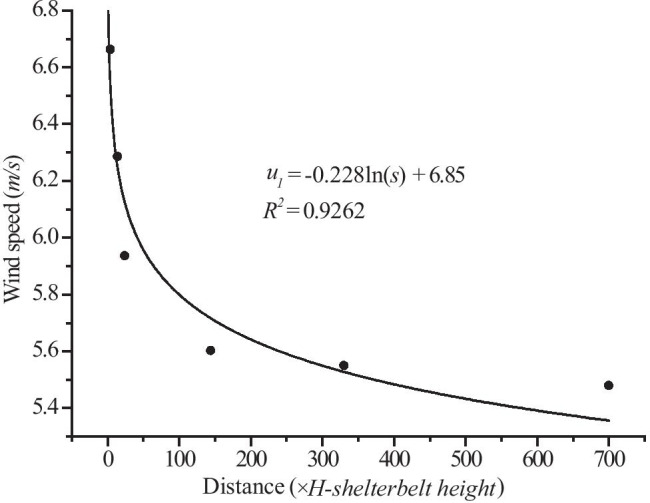
Wind speed reduction curve of the farm-shelter forest network

The farm-shelter forest network was investigated in the study area of Farm 224, Hotan Prefecture. The results revealed that the height of the shelterbelt was 10 m, the length of the principal shelterbelt was 500 m, the length of secondary shelterbelt was 100 m, the principal shelterbelt was perpendicular to the wind direction, the height of wind speed observation was 2 m, and the roughness was 0.003 m in the wilderness (Guan et al., 2001). The wind speeds were not high in the wilderness, and the wind in the farm-shelter forest network was very low due to the blocking effect of the shelterbelts in the study area. Therefore, high wind events were selected for our investigation, in order to better reflect the protective effect of the shelterbelts (Fig. 5). The high winds occurring in the study area on July 15 were selected, and the wind profile in the shelterbelts is plotted in Fig. 5.

Here, *u* is the wind speed, and *s* is the shelterbelt area (since the shelterbelt width is constant, *s* is correlated with the shelterbelt length, in units of *H*). The wind speed reduction curve in Fig. 5 shows that the velocity of the airflow decreased after passing through the first shelterbelt in the farm-shelter forest network. The velocity had not yet recovered to its initial speed when the flow reached the second shelterbelt. Therefore, the wind speed was very low, and eventually stabilized in the farm-shelter forest network (Maki, 1982; Cao, 1983; Bao et al., 2020). These results provided a database for the construction of the windbreak effect model used in this study, since the momentum flux was transmitted from the upper layer to the lower layer in order to maintain wind speed stability in the model.

### Wind profile of a single shelterbelt and the farm-shelter forest network for different wind speeds

**Fig. 6:**
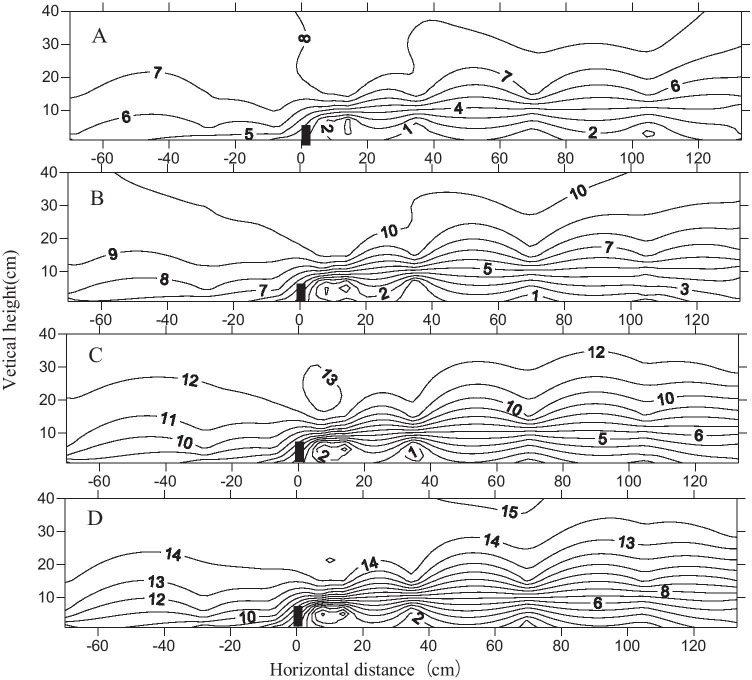
Flow field map of 8 rows of shelterbelt for different wind speeds (A: 8 m/s; B: 10 m/s; C: 12 m/s; D: 14 m/s)

**Fig. 7:**
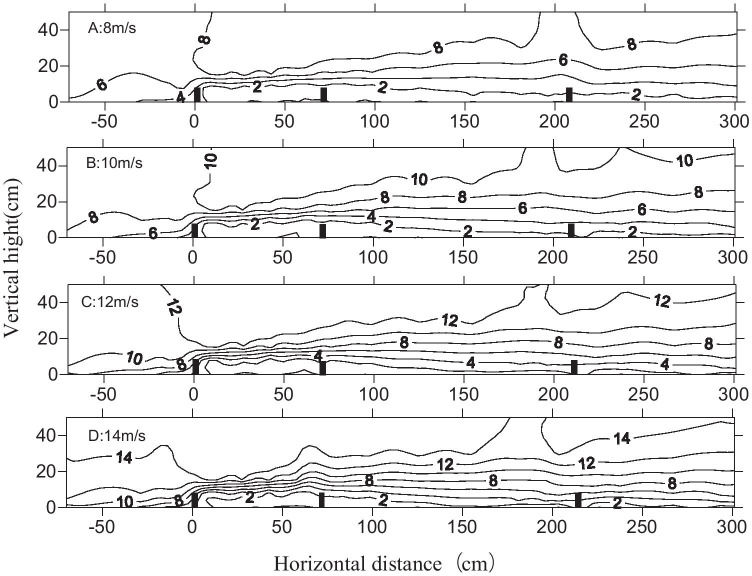
Flow map of shelterbelts for different wind speeds (A: 8 m/s; B: 10 m/s; C: 12 m/s; D: 14 m/s)

The wind flow field behind the shelterbelts had less impact for different wind speeds. The contours were tighter at the top of the canopy at high wind speeds, and there were more eddies at the back of the shelterbelts (Fig. 6). Upwind, the maximum wind decay distance was 9 *H*, when the wind speed reached 12 m/s; it was 7 *H* for wind speeds of 8 m/s and 10 m/s. Thus, wind speed was not the main factor responsible for changing the flow field shape. Wind decay was more pronounced in the farm-shelter forest network, and the wind speed maintained a relatively low level (Fig. 7).

### Comparing the measured results and the model results of the windbreak effects of the farm-shelter forest network

**Fig. 8:**
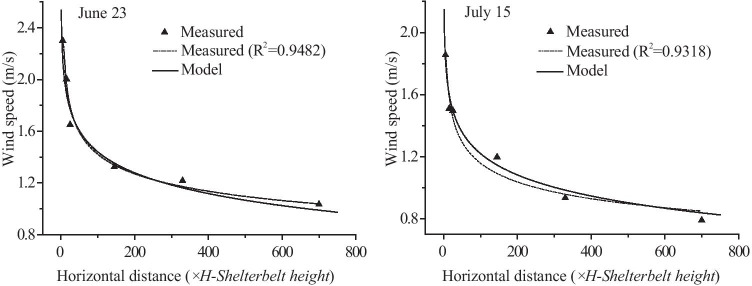
Wind speeds from the model and from actual measurements in different areas of the farm-shelter forest network

**Fig. 9:**
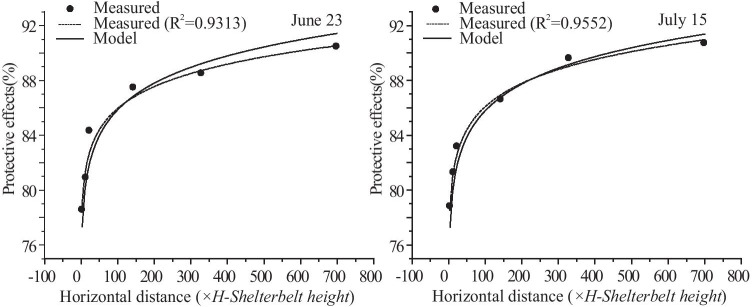
Protective effects from the model and from actual measurements in different areas of the farm-shelter forest network

Figure 8 shows a comparison of the model predictions with the actual measurement data. As can be seen from the Figs. 8 and 9, the results indicated that the model predictions were accurate. The analysis of the gales occurring on June 23 and July 15 had errors of < 1%, with the exception of the errors of 2.12% at 25 *H* and 1.3% at 700 *H* on June 23. The two relatively large errors on June 23 may have been the result of topographic fluctuations in the field. The windbreak effect increased with increasing forest area, strengthening 15% from 5 to 700 *H*. This increase reflected the cumulative effect of the windbreak in the farm-shelter forest network.

### Effect of the target jujube crop on the wind velocity flow field and the windbreak effect of the farm-shelter forest network in a wind tunnel

Since most of the shelterbelts in southern Xinjiang consist of 4 rows of trees, the 4-row shelterbelt (porosity: 30.12%) was selected for the wind tunnel experiments. Four open-field wind speeds were used: 8 m/s, 10 m/s, 12 m/s, and 14 m/s. The characteristics of the wind speed variations in front of and behind the shelterbelts were analyzed with and without the jujube trees in order to determine the effects of jujube on wind speed variations for different wind speeds in the farm-shelter forest network.

**Fig. 10:**
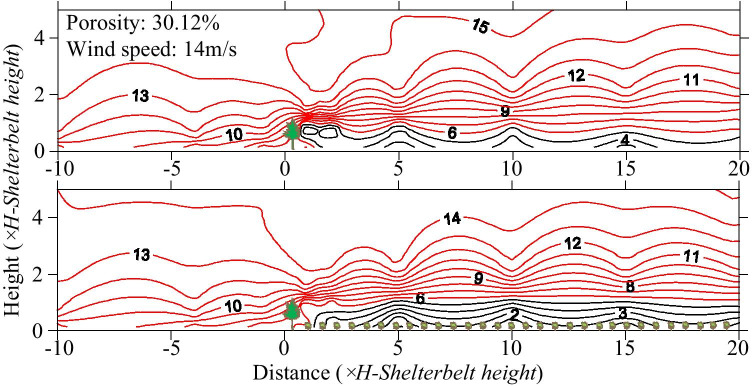
Wind speed contours with and without jujube

The results of the wind tunnel tests revealed that jujube significantly impacted the wind speed variations in front of and behind the shelterbelt (Fig. 10). The effect of jujube on the airflow lift in front of the shelterbelt was obvious for the same initial wind speed. The wind speed noticeably decreased behind the shelterbelt after the air had flowed through it. This effect was more pronounced when the jujube trees were present. At a height of 3.5 cm, the wind speed was reduced to 4 m/s in the shelterbelt without jujube trees, while the wind speed was reduced to 2 m/s in the shelterbelt with jujube trees. At a height of 3.5–7.0 cm, the wind speed was reduced to 5–7 m/s in the shelterbelt without jujube trees. With jujube trees, the wind speed was reduced to 3–5 m/s, and varied gradually with the horizontal gradient. The wind speed decreased significantly with the horizontal gradient above the height of the shelterbelt (7 cm), and the effect of jujube on the wind speed decreased with increasing height. The wind speed increased gradually until it reached its initial strength more than 5 *H* behind the shelterbelt, and slowly rebounded under the influence of the jujube trees.

**Fig. 11:**
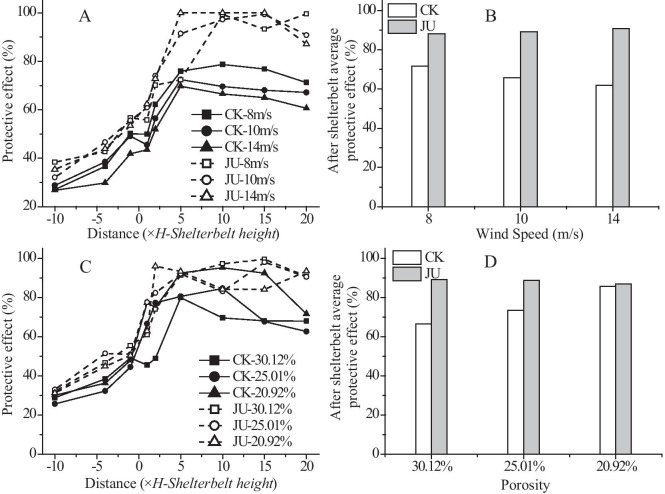
Protective effect of the shelterbelt for different wind speeds and porosity levels

The average windbreak effect at 2 different heights (1 cm and 2 cm) were selected as measures of the shelterbelt construction, since the main purpose of the shelterbelt was to protect the crops in the fields, and the crops were no taller than 3 m. As shown in Fig. 11A and B, the windbreak effect varied greatly with the horizontal gradient for different wind speeds and the same porosity. Jujube noticeably enhanced the windbreak effect of the shelterbelt, especially after the airflow had passed through the shelterbelt. The windbreak effect fluctuated a small amount from 2 to 20 *H* behind the shelterbelt, maintaining a level between 85 and 95%. There were obvious differences among the windbreak effects on the horizontal gradient of shelterbelts with different porosities. The windbreak effect of each shelterbelt increased significantly in the − 1 *H* to 5 *H* range. The increase of the windbreak effect of the shelterbelts with jujube trees was 35.6%, 41%, and 42.8% for porosities of 30.12%, 25.01%, and 20.92%, respectively. The increase of wind resistance of the shelterbelts without jujube trees was 30.8%, 36.1%, and 44% for porosities of 30.12%, 25.01%, and 20.92%, respectively. Between 1 and 20 *H*, jujube increased the windbreak effects of the shelterbelts with different porosities by 22.72%, 15.22%, and 1.28%, respectively.

Tang et al. (2012) showed that the windbreak effect of a single-row shelterbelt would decrease with increasing wind speed. Gao et al. (2010) showed that 1-m-high shrubs had a good windbreak effect in wind tunnel simulation experiments. In this study, jujube trees exhibited a similar windbreak effect, which played a synergistic role with the shelterbelts to effectively improve the overall windbreak effect. When the shelterbelt porosity increased, the horizontal roughness produced by the height and planting area of the jujube was synergistic with the windbreak effect. Thus, the effect of the jujube compensated for the decreased windbreak effect of more porous shelterbelts. Therefore, the windbreak effect of the shelterbelts with jujube trees was less affected by porosity.

### The optimal spacing interval between principal shelterbelts in the farm-shelter forest network

**Fig. 12:**
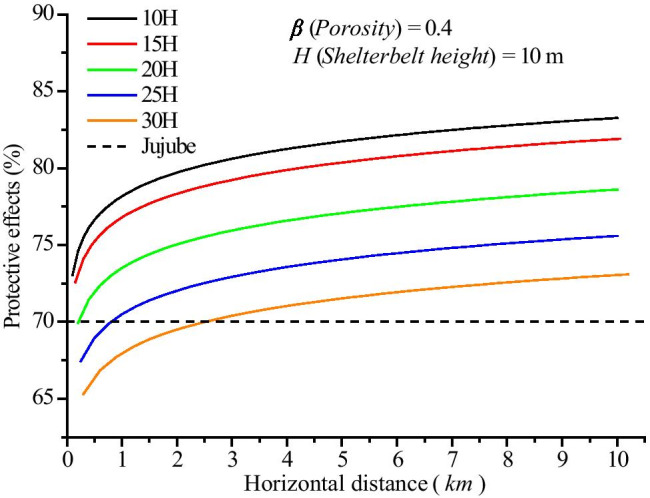
Protective effects of the farm-shelter forest network for different spacing intervals between principal shelterbelts. Dotted line: Protective effects when the wind speed in the wilderness outside the farm-shelter forest network reached 25 m/s and the wind speed in the farm-shelter forest network reached the damage threshold wind speed for jujube of 6.9 m/s (Zhu et al., 2016)

In this study, the shelterbelts of jujube farmland in the southern Tarim Basin of Xinjiang, China, were selected for model calculation. Field investigations revealed that the spacing interval between principal shelterbelts was 10 *H*, and there were 3 rows of shelterbelts in every 500-m field. Each shelterbelt had 2 lines of poplar, spaced 1.8 m apart. The windbreak effect decreased with increasing spacing interval. Specifically, in the farm-shelter forest network, the windbreak effect was reduced by 2.5% for every ~ 5 *H* increase in the spacing interval, with the exception of 15 *H*, at which the windbreak effect was reduced by ~ 1% (Fig. 12). Analysis of the threshold represented by the dotted line in the figure revealed that when the spacing interval was between 10 and 20 *H*, the shelterbelts could protect the jujube trees from winds up to 25 m/s. Meanwhile, the results also showed that when the spacing interval was 20 *H*, the crops in front of the farm-shelter forest network were affected by the wind, but almost all of the crops were protected by the shelterbelts. Therefore, due to its advantages, the spacing interval of 20 *H* was selected for use in actual production.

## Discussion

Wind tunnel experiments on a single shelterbelt and multiple shelterbelts were compared in this study. The results showed a pronounced difference between the wind speed profile and effective protection distance of a single shelterbelt versus the corresponding characteristics of multiple shelterbelts. We discovered that when the extreme wind speed in the wilderness outside the farm-shelter forest network exceeded 14–16 m/s, the wind speed experienced by the farmland was only about 4 m/s (Cao, 1983). This demonstrated that farmland shelterbelts can greatly reduce wind velocity, and this decrease in wind was due to the comprehensive effect of multiple shelterbelts (Li and Sherman, 2015; Bao et al., 2020). We found that the windbreak effect of the farm-shelter forest network was determined not only by the synergistic effect between shelterbelts, but also by the windbreak effects of tall target crops and the effect of humidity in the farm-shelter forest network. Considering the influence of the above factors on wind speed, a farmland shelterbelt allocation model was constructed based on momentum conservation. This model was then validated using the measurement data from two strong wind events. Our analysis demonstrated that reliable prediction results were obtained.

The effect of protected crops on wind speed has not attracted enough attention in previous research on shelterbelt construction. This study considered the influence of protected target crops on wind, primarily in the form of the humidity effect produced in extensive crop areas, and the increased surface friction resistance caused by high crops (e.g., the height of the jujube trees ranged from 1.5–4 m) in the farm-shelter forest network. Through model construction and numerical simulation analysis, it was discovered that the humidity effect of the target crops was relatively weak, but friction resistance was increased markedly by jujube. Wind tunnel experiments also showed that when target crops were present, the windbreak effect of shelterbelts was enhanced and the amplitude was greater. Therefore, the influence of target crops (especially fruit trees) on the windbreak effect cannot be ignored.

Farm-shelter forest network construction is determined not only by the spacing interval between principal shelterbelts (Zhu, 2013) but also by shelterbelt porosity (Guan et al., 2002; Van Thuyet et al., 2014; Zheng et al., 2016a, b; Sun et al., 2020). Through field observations, wind tunnel experiments, and numerical simulation, we found that the windbreak effect of the farm-shelter forest network was primarily affected by shelterbelt spacing and porosity. In this investigation, a common shelterbelt porosity value (*β* = 0.4) was selected for the model that was designed to determine farmland shelterbelt allocation and construction. However, the number of rows, row spacing, and other structural characteristics affecting porosity also need to be considered in the actual process of shelterbelt construction. Following the determination of the optimal porosity, the spacing interval between principal shelterbelts was quantified, based on the target crop requirements and shelterbelt species.

A large number of studies have shown that the pattern of “narrow shelterbelt (maintaining a certain degree of porosity), small grid (a spacing interval between principal shelterbelts of about 10 *H*)” was adopted in farm-shelter forest network construction from the early 1960s to the present in areas of Xinjiang that experience serious sandstorms (Zhao et al., 2009). In this study, quantitative analysis demonstrated that when the spacing interval between principal shelterbelts increased from 10 to 20 *H*, more than 70% of the protective windbreak effect of the farm-shelter forest network could be maintained. This result indicates the feasibility of further reducing the spacing interval between principal shelterbelts and decreasing the shelterbelt areas, thereby enhancing the economic benefits of crops. For example, if the spacing interval between principal shelterbelts was increased from 10 to 20 *H* in the jujube fields of southern Xinjiang, the planting area would increase by 0.54% (2700 m^2^). The annual profit of jujube is currently $45,000 per hectare. Thus, the annual profits from the 470,000 ha of jujube trees in southern Xinjiang would increase by $113 million. In addition, expanding the spacing interval between principal shelterbelts would be conducive to agricultural mechanization, which would greatly improve production efficiency.

## Conclusions

The farm-shelter forest network is a complex grid protection system, with a windbreak effect that differs significantly from that of a single shelterbelt. The windbreak effect of a farm-shelter forest network is determined not only by the windbreak effect of each shelterbelt, but also by the synergistic effect among shelterbelts, the windbreak effects of tall target crops, and the effects of temperature and humidity. In this study, the spacing interval between principal shelterbelts was increased due to the windbreak effect of the farm-shelter forest network. Specifically, the spacing interval between principal shelterbelts was increased from 10 to 20 *H* in a jujube field of southern Xinjiang. This increase maintained the windbreak effect while reducing the side effects of the shelterbelts, increasing the planting area, and improving the planting efficiency. This change to the farm-shelter forest network construction pattern has the potential to significantly promote the development of modern intensive agriculture, precision agriculture, and mechanized agriculture.

## Data Availability

The datasets used and/or analyzed during the current study are available from the corresponding author on reasonable request.
